# Aesthetic Clinical Case for Immediate Implant Therapy for Maxillary Central Incisor

**DOI:** 10.1155/crid/4199406

**Published:** 2024-12-03

**Authors:** Gerardo Guzman, Silvia Rojas-Rueda, Franciele Floriani, Carlos A. Jurado, Francisco X. Apiazu-Flores, Nicholas G. Fischer

**Affiliations:** ^1^Department of Periodontology, Centro Educativo Multidisciplinario en Rehabilitacion Oral (CEMRO), Tarimbaro, Michoacan 58880, Mexico; ^2^Department of Periodontology, Quetzalcoatl University, Irapuato, Guanajuato 36615, Mexico; ^3^Dental Biomaterials, School of Dentistry, The University of Alabama at Birmingham, Birmingham, Alabama 35233, USA; ^4^Department of Prosthodontics, College of Dentistry and Dental Clinics, The University of Iowa, Iowa City, Iowa 52241, USA; ^5^Division of Operative Dentistry, Department of General Dentistry, College of Dentistry, The University of Tennessee Health Science Center, Memphis, Tennessee 38103, USA; ^6^Department of Restorative and Prosthetic Dentistry, The Ohio State University College of Dentistry, Columbus, Ohio, USA; ^7^Minnesota Dental Research Center for Biomaterials and Biomechanics, School of Dentistry, University of Minnesota, Minneapolis, Minnesota 55455, USA

**Keywords:** dental implants, diagnostic, aesthetic, imaging, immediate implants

## Abstract

This case report features a female patient with the chief complaint of needing to replace an anterior crown. After a comprehensive oral assessment and cone beam computed tomography (CBCT) radiographic examination, it was determined that the crown on Tooth #9 was subgingivally fractured. The procedure involved atraumatic extraction of Tooth #9, followed by immediate implant placement. Xenograft bone graft material was placed to complete the space between the buccal bone and the implant. A connective tissue graft (CTG), 1 mm thick and 5 mm wide, was harvested from the palate and placed. The final implant crown was restored using a prefabricated abutment with a titanium base and zirconia ceramic dental material. A well-planned combined treatment, including atraumatic tooth extractions for immediate implants and ideal contouring of soft tissues, can significantly impact the outcome of aesthetic restorations. A single immediate implant-supported crown in the aesthetic zone was able to fulfill the patient's aesthetic expectations at the 2-year follow-up.

## 1. Introduction

Immediate implant placement has gained popularity among dentists and patients as a desirable treatment option. This approach not only shortens the overall treatment duration and reduces clinical morbidity but also plays a crucial role in preserving peri-implant mucosal tissue following tooth extraction [1]. However, achieving the ideal three-dimensional positioning of the implant while ensuring primary stability presents challenges, largely due to factors such as the morphology of the extraction socket and the surrounding alveolar bone [2].

Despite its advantages, immediate implant placement does not inherently limit alveolar bone resorption, which typically accompanies tooth extraction [3]. Ridge alterations and bone remodeling processes are primarily observed within the first 12 months postextraction [4]. However, an increase in soft tissue remodeling, as reflected by the pink aesthetic score (PES), suggests that although bone modifications occur, they may not be clinically evident when managed with proper clinical case selection and appropriate hard and soft tissue grafting procedures [5, 6]. In the realm of immediate implant placement, the role of adjunctive surgical procedures becomes pivotal for ensuring long-term success rates [7]. These procedures vary, ranging from flapless to open-flap surgical approaches, and involve different bone graft materials such as autologous, allograft, or xenograft [8]. Additionally, the type of connective tissue graft (CTG) used significantly impacts clinical decision-making and treatment outcomes [9].

The adoption of immediate implant placement in the aesthetic zone necessitates careful consideration of several pre- and intraoperative factors to minimize aesthetic compromise and maximize overall success. Over the past two decades, extensive research on immediate implant placement in partially edentulous patients has demonstrated survival rates comparable to those of delayed implant placement using conventional surgical approaches [10–12]. A previous systematic review assessing the influence of an immediate implant protocol on clinical performance and aesthetic outcomes revealed an overall implant survival rate up to 5 years of 95.8% (93.3%–97.4%), which aligns with survival rates for other implant placement concepts, and a restoration survival rate after 5 years of 94.8% [7]. Additionally, 10-year data on the outcomes of fixed implant-supported restorations showed a survival rate of 95.5%, with a 98.8% implant survival rate noted in the literature [13]. No significant influence of retention type (screw vs. cement) on the survival rate was observed [14].

A multidisciplinary approach that integrates immediate implant planning with prosthodontics is crucial for achieving long-term functional and aesthetic success. However, the challenge of combining tooth-supported crowns with a single immediate implant-supported crown in the aesthetic zone poses significant risks for aesthetic complications. Recently, monolithic and layered zirconia crowns have become increasingly popular for anterior crowns, representing 84% of treatments in a material selection survey conducted by the National Dental Practice-Based Research Network [15]. These crowns are designed to provide high-strength restorations with an adequate clinical appearance [16]. This case report describes a conservative approach involving the atraumatic extraction of a maxillary central incisor and its replacement with a single immediate implant placed in a fully guided surgery, highlighting the use of zirconia ceramics in the aesthetic zone for restoring single immediate implant-supported crowns over a follow-up period of 2 years.

### 1.1. Clinical Considerations

Multiple tools have been introduced to evaluate aesthetic outcomes in immediate implant placement (Table 1). The following clinical conditions are recommended when planning for immediate implant placement:

• Intact socket bone walls: The condition of the facial bone wall is crucial for aesthetic outcomes. Diagnostic cone beam computed tomography (CBCT) scans are commonly used to assess the integrity of the facial socket wall.
• Thick soft tissue phenotype: Sites with thin soft tissue carry a higher risk of recession of the midfacial mucosa, potentially negatively affecting the final aesthetic outcomes.
• No acute infection at the site: Sites exhibiting acute infection should not be considered for immediate implant placement, as inflammation can lead to significant recession.
• Sufficient bone volume apical and lingual to the socket: Adequate bone volume ensures that the implant can be placed with primary stability.

### 1.2. Aesthetic Scores

• PES: evaluates the peri-implant mucosa using seven specific soft tissue parameters, including the presence or absence of mesial and distal papillae, level of the facial mucosal margin, soft tissue contour, alveolar process deficiency (facial convexity), soft tissue color, and texture. Each parameter is scored from 0 to 2, with 2 being the best score and 0 the poorest, for a total possible score of 14.
• White aesthetic score (WES): focuses on the aesthetic evaluation of an implant restoration based on five parameters: tooth form, outline, color, surface texture, and translucency. Each parameter also receives a score between 0 and 2, for a maximum possible score of 10.
• Aesthetic risk assessment (ERA): designed for the treatment of partially edentulous patients with dental implants, the ERA aids clinicians in diagnosing and planning treatment in the aesthetic zone and identifying clinical factors or situations that could lead to aesthetic compromise.

## 2. Materials and Methods

**Figure 1:**
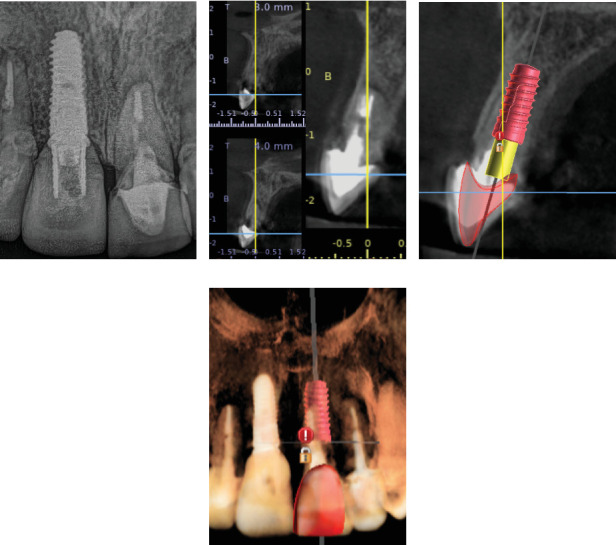
Initial imaging assessment. (a) Initial radiograph, (b) CBCT evaluation, (c) implant planning sectional view, and (d) implant planning frontal view.

A 45-year-old female patient presented to the clinic with the chief complaint of mobility in the upper front tooth and had porcelain fused to metal (PFM) crown. Radiographic and CBCT evaluation revealed a fracture of the clinical crown and was diagnosed as hopeless. A digital evaluation was performed for implant therapy (Figure 1).

**Figure 2:**
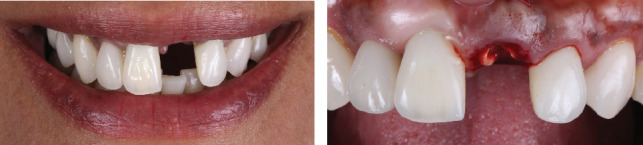
Fractured tooth. (a) Smile with a fractured tooth and (b) intraoral view of a fractured tooth.

The patient was informed that implant therapy was an option along with other options, including a fixed dental prosthesis from the right central incisor to the left lateral incisor and a removable partial denture as well. The patient selected implant therapy. During the occlusion evaluation, the clinical crown came off, and the patient requested to start the treatment as soon as possible (Figure 2).

Clinical conditions evaluated to finalize the treatment plan included socket bone walls, soft tissue phenotype, no signs of infection, PES and WES, and ERA to evaluate the possibility of extraction and immediate implant (Table 2). The treatment option consisted of the replacement of the upper left central incisor with a single immediate implant placement and immediate loading of a single supported crown with CTGs for buccal volume augmentation and to rectify the midfacial recession, and bone graft with allograft material was also proposed.

**Figure 3:**
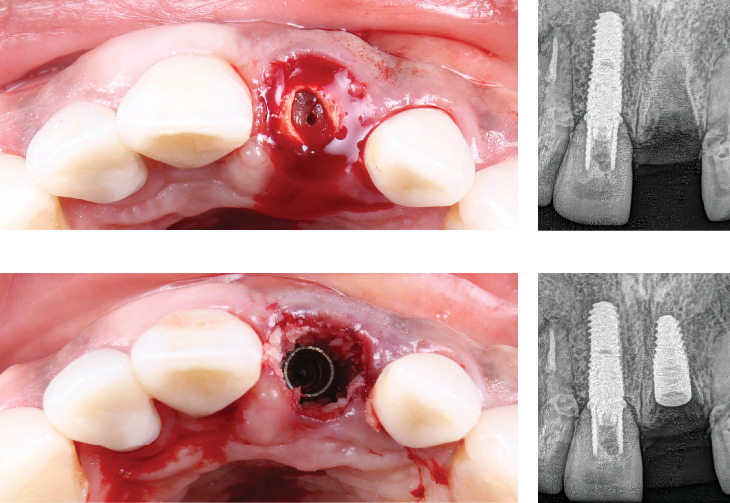
Extraction and implant placement. (a) Fractured tooth occlusal view, (b) radiograph of extracted tooth, (c) implant placement occlusal view, and (d) radiograph of implant placement.

Digital planning for tooth extraction and immediate implant placement was managed using CBCT radiography, and the thickness of the buccal and lingual bone allowed the conventional placement of an implant with a 10 mm length and 4.1 mm diameter. Atraumatic flapless tooth extraction was conducted with piezoelectric instruments (Piezomed, W&H, Bürmoos, Austria) in order to preserve the intact buccal. The surgical guide was positioned, and an implant 10 mm × 4.1 mm (Bone Level Tapered, Straumann, Basel, Switzerland) was drilled for immediate implant placement with palatal direction to avoid injury to the labial bone plate and to provide a room between the outer surface of the implant fixture and the inner surface of the labial plate. The jumping distance ranged from 2 to 3 mm, which was determined by controlling the implant diameter. Bone graft material xenograft bone (Geistlich Bio-Oss; Pharma AG, Bahnhofstrasse 40 CH-Wolhusen) was used to complete the space between buccal bone and implant (Figure 3).

**Figure 4:**
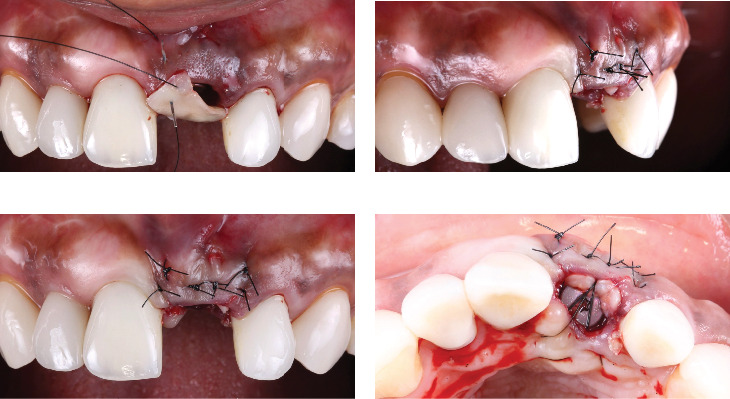
CTG placement and suturing. (a) Frontal view during suturing, (b) lateral view after suturing, (c) frontal view after suturing, and (d) occlusal view after suturing.

A CTG (1 mm thick and 5 mm wide) was harvested from the palate after local anesthesia with 40mg/mL001mg/mL of articaine. The length of the CTG was equal to that of the site to reconstruct measured from the buccal aspect. The CTG was tunneled into a pouch with a microblade between the keratinized mucosa and the bone graft material/cortical plate of the socket. Interrupted sutures in the mesial and distal aspects of the sockets were used to stabilize the CTG in position (Figure 4).

**Figure 5:**
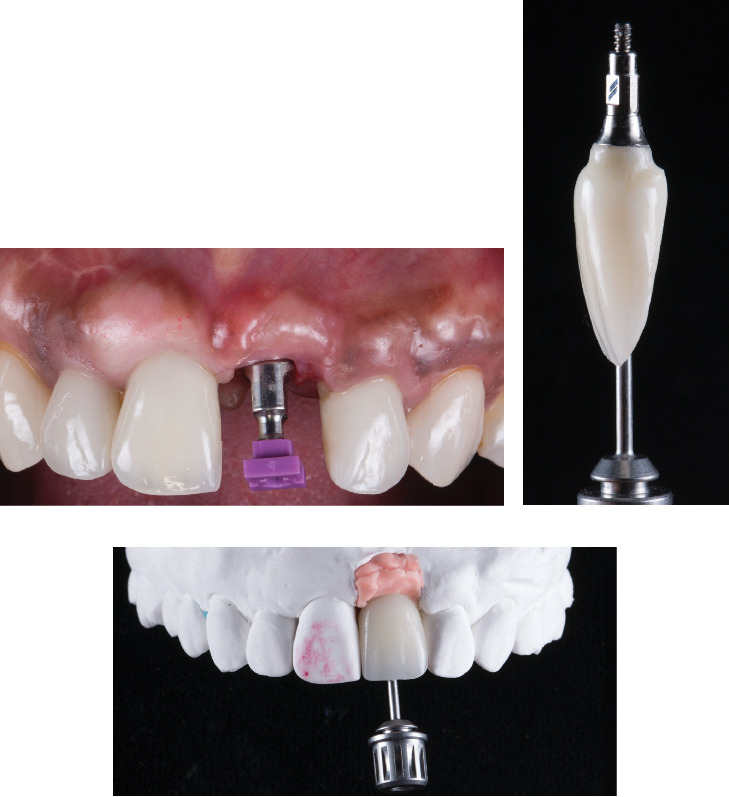
Final impression and restoration. (a) Impression post in mouth, (b) fabricated screw-retained implant crown, and (c) restoration in master cast.

The healing process of the soft tissue was monitored, and at 4 months after implant placement, the provisional approach was modified to include single crowns and screw-retained provisional restoration. The final implant impression (RC Impression Post, Straumann Group, Basel, Switzerland) of the teeth and implant were taken. Subsequently, a screw-retained monolithic zirconia implant-supported crown was fabricated (Figure 5).

**Figure 6:**
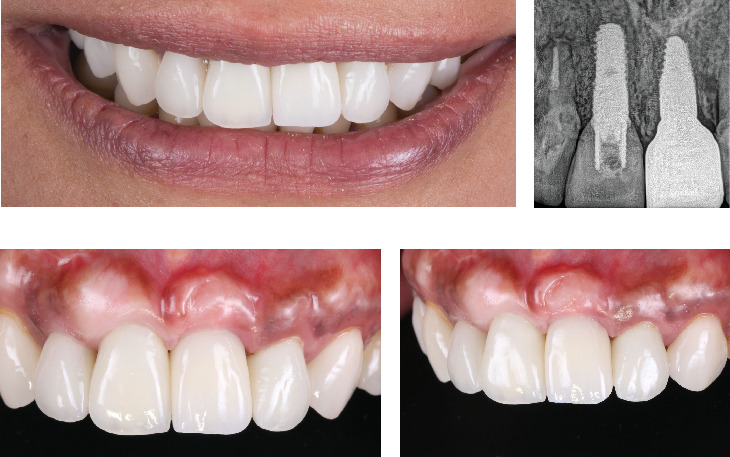
Final restoration in the mouth. (a) Final smile, (b) radiograph, (c) final frontal view, and (d) final left side view.

The final screw-retained implant restoration was placed in the mouth, and the access hole was sealed with Teflon tape and flowable composite (Figure 6).

Occlusion, maximum intercuspation, excursive movements, and protrusion were checked. The patient was pleased with the shade and shape of the final restorations. A full-mouth guard was provided to protect the restorations.

## 3. Results

A multidisciplinary approach was integral to the treatment plan, considering factors provided by PES, WES, and ERA, such as buccal bone presence and tissue biotype, to guide the decision between early or delayed implant placement. The plan also addressed aesthetic concerns, including maintaining the interproximal papilla between the implant and natural teeth, distributing interproximal space between upper incisors, and achieving color-blending with different structures (tooth and implant).

Digital implant planning played a crucial role in ensuring accuracy in the 3D implant position. Emphasis was placed on implant depth to provide an adequate emergence profile and achieve gingival symmetry between the implant and natural teeth. Immediate implant placement was executed, and to prevent complications from both biological and aesthetic perspectives, CTGs were employed to enhance tissue augmentation. Factors such as interproximal attachment of adjacent teeth, the presence of bone on the buccal surface of the implant, and horizontal and vertical implant positions were considered.

## 4. Discussion

Achieving aesthetic outcomes by integrating single immediate implant-supported crowns in the aesthetic zone presents a complex challenge compared to utilizing a singular type of restoration. The timing of implant placement relative to tooth extraction can be categorized into delayed, immediate-delayed post-soft tissue healing, and immediate placement which typically preserves the extraction socket walls [19, 20]. Prior studies have indicated that immediate implant placement carries a 20%–30% higher risk of mucosal recession compared to other protocols [21, 22]. To mitigate this risk, atraumatic tooth extraction followed by connective and hard tissue grafting is employed to maintain gingival contours.

Atraumatic extraction combined with immediate implant placement has been observed to harmonize aesthetics and the health of keratinized tissue [23], primarily by preserving the labial bone plate during extraction and maintaining intact mucosa without employing an open flap technique. When an implant is optimally placed three-dimensionally within the bone, a gap often emerges between the implant's outer surface and the labial plate's inner surface, referred to as the “jumping gap.” One study examining the histologic outcomes of a substantial horizontal jumping gap of 4.2 mm without bone grafting showed direct bone contact up to the last implant fixture thread, suggesting that immediate implantation in sockets with intact buccal bone can achieve osseointegration without bone grafts [24]. In our study, the osteotomy for immediate implant placement was strategically oriented palatally to avoid damaging the labial bone plate, allowing for a controlled jumping gap of 2–3 mm, which we filled up to the bone level with graft material, extending to the soft tissue margin. Immediate implant placement posttooth extraction demonstrated high survival rates and favorable aesthetic results, significantly appealing to both clinicians and patients. A systematic review assessing immediate postextraction implants reported a 98.4% success rate at 2 years, with marginal bone loss under 1 mm [25]. Similarly, a clinical trial comparing immediate and delayed implant placement over 5 years found no significant differences in failure rates between the maxilla and mandible, with success rates of 92.4% and 94.7%, respectively [23].

Choosing between immediate or delayed placement often hinges on the treatment's impact on the patient since long-term survival rates are generally comparable. The patient in our case study opted for immediate implant placement to minimize surgical interventions and save time.

Furthermore, the CTG technique, aimed at minimizing peri-implant tissue changes, has shown successful functional and aesthetic outcomes, particularly evident in a randomized clinical trial that assessed soft tissue augmentation during implant placement in patients with a thin gingival phenotype. The results demonstrated excellent aesthetics as per the PES up to 12 months postrestoration [24–27].

In this case report, the implant was positioned using computer-designed surgical guides based on a prosthetically driven implant protocol, ensuring optimal placement that minimizes risks such as facial mucosal margin recession and maximizes aesthetic outcomes. Our study's findings advocate for immediate implant placement whenever bone dimensions allow, as it significantly improves both PES and WES, maintaining an optimal emergence profile with a polished temporary crown. Immediate implants after maxillary anterior tooth extraction have proven to achieve satisfactory aesthetic outcomes.

This study's limitations include the necessity to investigate the most effective immediate implant protocols in patients with defective labial bone plates and to elucidate the relationship between long-term mucosal stability and the position of the facial bone crest. Future research should delve into the interaction between mucosal stability, the type of bone graft material used, and the facial bone's position and thickness.

## 5. Conclusions

Atraumatic tooth extractions for immediate implants and ideal contouring of the soft tissues can impact the outcome of the result and fulfill the patient's aesthetic expectations. Here, we showed a single immediate implant-supported crown in the aesthetic zone was able to fulfill the patient's aesthetic expectations at the 2-year follow-up.

## Figures and Tables

**Table 1:** Aesthetic risk assessment (ERA) table outlining the factors that can be assessed to determine the level of aesthetic risk associated with implant tooth replacements irrespective of the protocol for implant placement or loading [17].

**Aesthetic factor**	**Level of risk**	
	**Low**	**Medium**	**High**
Medical status	Healthy, uneventful healing		Compromised healing
Smoking habit	Nonsmoker	Light smoker (≤ 10 cigarettes/day)	Heavy smoker (> 10 cigarettes/day)
Gingival display at full smile	Low	Medium	High
Width of edentulous span	1 tooth (≥ 7 mm) 1 tooth (≥ 6 mm)	1 tooth (≥ 7 mm) 1 tooth (≥ 6 mm)	2 teeth or more
Shape of tooth crowns	Rectangular		Triangular
Restorative status of neighboring teeth	Virgin		Restored
Gingival phenotype	Low-scalloped, thick	Medium-scalloped, medium-thick	High-scalloped, thin
Infection at the implant site	None	Chronic	Acute
Soft tissue anatomy	Soft tissue intact		Soft tissue defects
Bone level at adjacent teeth	≤ 5 mm to contact point	5.5–6.5 mm to contact point	≥7 mm to contact point
Facial bone-wall phenotype	Thick-wall phenotype ≥ 1 mm thickness		Thin-wall phenotype < 1 mm thickness
Bone anatomy of alveolar crest	No bone deficiency	Horizontal bone deficiency	Vertical bone deficiency
Patient's aesthetic expectations	Realistic expectations		Unrealistic
			Expectations

**Table 2:** PES/WES evaluation according to Jones and Martin [18].

	**Absent**	**Incomplete**	**Complete**
PES			
Distal papilla	0	0	2
Mesial papilla	0	0	2
Curvature of facial mucosa	0	0	2
Level of facial mucosa	0	0	2
Root convexity/soft tissue color	0	0	2
Maximum total PES	0	0	10
WES			
Tooth form	0	0	2
Tooth volume/outline	0	0	2
Color	0	0	2
Surface texture	0	0	2
Translucency	0	0	2
Maximum total WES	0	0	10

## Data Availability

The data that support the findings of this study are openly available online: PubMed, Clinical Reports in Dentistry, and University of IOWA.
